# Accrual in supportive care trials in pediatric oncology, a challenge!

**DOI:** 10.1007/s00520-012-1447-2

**Published:** 2012-04-04

**Authors:** R. A. Schoot, C. H. van Ommen, H. N. Caron, W. J. E. Tissing, M. D. van de Wetering, SKION Aristocaths supportive care group the Netherlands

**Affiliations:** 1 Department of Pediatric Oncology, Emma Children’s Hospital, Academic Medical Center (AMC), University of Amsterdam, PO Box 22660, 1100 DD Amsterdam, The Netherlands; 2 Department of Pediatric Hematology, Emma Children’s Hospital, Academic Medical Center (AMC), University of Amsterdam, Amsterdam, The Netherlands; 3 Department of Pediatric oncology, Beatrix Children’s Hospital, University of Groningen, Groningen, The Netherlands; 4 Dutch Childhood Oncology Group, The Hague, The Netherlands

**Keywords:** Supportive care, Pediatric oncology, Accrual, Catheter-related infections, Patient enrolment

## Abstract

**Purpose:**

Treatment protocols in pediatric oncology have historically known high accrual rates, up to 94 %. Accrual for supportive care studies on the other hand appears to be a challenge. The aim of this study was to search for reasons explaining this poor accrual and for possible interventions to improve patient enrolment.

**Methods:**

The failure screen log of our supportive care study (the Aristocaths study) was analyzed, and subsequently, a literature search was performed.

**Results:**

The literature search (1985–2011) revealed three factors that can influence accrual. Firstly, study implementation and patient enrolment can be facilitated by appointing a dedicated clinical investigator in all participating centers and by facilitating clinical research nurses. Furthermore, adequate and tailor-made information is required for families to make a well-informed decision regarding study participation. Lastly, sufficient time should be assured for the process of decision making, especially since the number of eligible studies is increasing rapidly. Concerning our study, all three elements were met, but the most striking finding was the presumed burden of study participation by the majority of parents (82 %) as the main argument against randomization.

**Conclusions:**

Accrual of pediatric oncology patients in supportive care studies is challenging. Nevertheless, well-designed randomized controlled trials in supportive care will be essential for the improvement of pediatric cancer care. Therefore, we will need to increase awareness through (inter)national supportive care working groups regarding the need for supportive care trials and stimulate accrual when such trials are open.

## Introduction

Participation in clinical trials clearly benefits the outcome of oncology patients. Pediatric oncology has historically known high accrual rates for treatment protocols, and in the past decades, extensive research and inclusion in treatment protocols has led to an increase in 10-year survival from 10 to 80 % for children with cancer [1–4]. Accrual for other types of clinical trials on the other hand, such as nontherapeutic trials or supportive care trials, appears to be a challenge in the same patient population [5]. An explanation for this disparity might be the fact that these studies do not involve the cancer treatment itself but supportive care interventions needed during and after treatment. It is well known that at the start of treatment, both parents and the child receive a lot of information on the treatment and complications that can occur. This causes emotional distress making it difficult to realize what is coming. In the current pediatric oncology practice, the child is eligible to more than one study at the same time. In our center for example, children diagnosed with acute lymphoblastic leukemia are eligible to five different studies: one treatment protocol and four supportive care or non-treatment protocols. Because decisions are often required within the first few days after diagnosis, some studies are mentioned in the same consultation as the actual diagnosis. All studies are approved for by the medical ethical committee. All are clinically relevant and aimed to improve cancer treatment. But how much information can one handle? Which study has priority?

To gain insight in the reasons for the poor accrual in supportive care studies, we analyzed the failure screen log of our supportive care study: the Aristocaths. Since the start of this study, we have registered reasons to refuse study participation in the failure screen log. Subsequently, we performed a literature search to search for reasons explaining this accrual rate and focus on points of improvement.

## The Aristocaths study

The Aristocaths study (NTR 1275) started in The Netherlands in 2007. This study investigates the role of 70 % ethanol locks in the prevention of catheter-related infections in pediatric cancer patients with tunneled central venous catheters (CVC) [6]. However, over the past 3 years, accrual turned out to be lower than expected, i.e., about 50 % of the projected accrual.

### Failure screen log

Since the start of the Aristocaths study, eligible patients were registered at all four participating study sites. If patients were eligible but decided not to participate, the reason for their refusal was registered in the failure screen log. The study is still ongoing; failures reported in this study have been registered between October 2007 and December 2010.

**Fig. 1:**
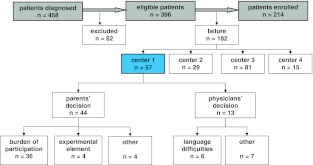
Flowchart of patient selection. Analysis of patients diagnosed between 2007 and 2010 in four participating centers and registered in the failure screen log. Full text registrations were kept for the initiating center (nr. 1) only

Between October 2007 and December 2010, 458 patients were diagnosed in the four participating pediatric oncology centers (Fig. 1). Sixty-two patients (14 %) were excluded for the following reasons: (1) age less than 1 year, (2) documented infection at the time of catheter insertion, (3) a previous central venous catheter, or (4) because study introduction took longer than a month. Since most catheter-related infections occur within 45 days, randomization and placement of the first study lock shortly after CVC placement is aimed for.

Considerations not to participate were grouped as “parents’ decision” or “physicians’ decision.” Failure rates ranged between 37 and 61 %, with the lowest rate in the initiating center (Table 1). In this center (Table 1, center nr. 1), these considerations were registered with a full text description of the reason for refusal. In total, 57/182 failures (31 %) occurred in this center. In 44/57 (77 %) failures, the decision not to participate in the Aristocaths study was made by the parents. Main argument for 36/44 (82 %) families was the potential burden of study participation. This burden was either explained as an actual time burden; 2-h waiting for the study lock to take place, or as a psychological burden; to take in all new information and make a decision. Parents expressed the following considerations: “everything is too much,” “2-h waiting is too much,” and “fear for CVC manipulation.” Four other families were troubled by the experimental aspect of participation: “do not want my child to be used as a guinea pig.” Other considerations were: “evidence unconvincing,” “enough other children to participate,” or “risky, side effects are unclear.” In 13/57 (23 %) failures, the treating oncologist decided not to ask the family for informed consent: most often because of language difficulties (six patients, 46 %). Other reasons for treating oncologists to refrain from study introduction were: “palliative treatment,” “physical condition of the child,” “complicated treatment history,” and “autistic disorder.”

**Table 1:** Number of eligible patients, number of patients excluded from randomization and failure rates, specified per participating center

Center	Eligible	Excluded	Failure
	*N*	*N* (%)	*N* (%)
1.	167	12/167 (7.2)	57/155 (37)
2.	88	15/88 (17)	29/73 (40)
3.	162	30/162 (19)	81/132 (61)
4.	41	5/41 (12)	15/36 (42)
Total	458	62/458 (14)	182/396 (46)

## Failure analysis

Patient accrual in randomized clinical trials is a multifactorial process. Several studies have described factors influencing accrual rates and employed the following classification: trial factors, physicians’ perspectives, parents’ perspectives, and child factors [1, 4, 7]. This classification was used for evaluation of factors affecting the accrual rate of the Aristocaths study.

### Trial factors

The Aristocaths study is a national multicenter pediatric oncology study in supportive care and is supported by the Dutch Childhood Oncology Group, thereby prescribing study participation nationwide. Yearly, approximately 600 newly diagnosed patients are treated in The Netherlands. With an estimated accrual rate of 50 %, randomization of 200 patients in both arms was calculated to be easily achieved within 3 years. Unfortunately, understaffing of research facilitating personnel and organizational issues have made implementation of the Aristocaths study impossible for two out of six oncology centers, thereby leading to smaller numbers of eligible patients and longer study duration. This can in part be attributed to the study itself; implementation of the Aristocaths study is labor intensive and requires a lot of effort by the clinical research team, constantly monitoring the study patients for lock eligibility and the occurrence of infectious or serious adverse events. Limited staff and funding are common problems, especially for supportive care studies such as the Aristocaths study since these studies are given lower priority than therapeutic protocols [5].

Since the incidence of cancer in children is rare, national and international cooperation in multicenter trials is required. Such cooperation can be a logistic and methodological challenge: to obtain approval from different medical ethical committees, to organize research facilitating personnel, to conform to good clinical practice legislation, and to motivate clinicians about the relevance of the research question. Carter et al. reported successful interventions in nontherapeutic Childhood Oncology Group studies, such as the implementation of coordinating centers, principal investigators and monetary reimbursements, leading to improved study execution and patient enrolment [5]. Although the abovementioned interventions were realized for the Aristocaths study, accrual remained poor because less centers were involved in this study as intended.

### Physicians’ perspectives

Disbelief in the benefit of study participation by treating physicians has been mentioned one of the major barriers in adult medicine [8–10]. Caldwell et al. investigated the attitudes of 21 pediatric physicians towards randomized controlled trials in children. Physicians mentioned feeling uncomfortable with the chance of randomization to the placebo arm, particularly for terminally ill patients. Others thought disclosure of uncertainty in regard to the best available treatment would lead to mistrust by parents, expecting their doctor to know all the answers [11].

In case of the Aristocaths study, the 50 % chance of being randomized to the placebo arm with 2-h waiting without treatment benefits was a strong argument for both physicians and families against study participation.

### Parents’ perspectives

Parents are responsible to decide upon trial participation in their child’s best interest, with or without the assent of the child itself. A truly informed decision is difficult and is even considered impossible by some [12]. The key ingredient for such decisions is the availability of adequate information, both by personal communication and in writing [13]. Nevertheless, Kodish et al. reported that despite oral and written explanation, half of the parents in their cohort did not understand the principle of randomization. They observed explanations of randomization and parental understanding afterwards in 137 informed consent consultations. Although 83 % of physicians explained the principle of randomization, 50 % of parents did not comprehend this key aspect of their decision [14]. Partly, this knowledge gap can be attributed to the emotional distress parents experience, confining them from taking in new and complex information. Furthermore, patient informed consent forms (PIFs) often contain too many legal details, required by good clinical practice guidelines, which makes the PIFs unreadable. What is the optimal approach for study introduction and how can adequate information be delivered? Eder et al. interviewed 140 parents of children who had been offered participation in a randomized clinical trial for the treatment of their acute leukemia. In this study, parents expressed the desire for sufficient time to make a decision and to consult others. Furthermore, information should be tailor made and adjusted to parents’ desires [15]. Parents also advocated the need for repetition, honest and empathic communication, with limited jargon and plenty of opportunity and encouragement to ask questions [13].

### Child factors

Child factors differ among age groups: being infants, (young) children, or young adults. For the first two groups, decisions concerning treatment are predominantly made by parents. In the group of adolescents and young adults on the other hand, decisions are made by the young adult himself or with the adolescent’s assent. This group, however, represents the lowest rate of trial participation: Ferrari et al. reported in 2008 that rates in the age group of 15 to 19 year olds were lower than in any other 5-year age group below or above age 35 [16]. Apart from lower trial participation among adolescents and young adults in general, these patients often receive their cancer treatment in medical oncology centers, where accrual rates are lower and clinicians are less familiar with pediatric protocols [17]. This does not account for the Aristocaths study: in The Netherlands, all pediatric oncology patients (until the age of 18) are treated in pediatric oncology centers and thus eligible for study participation. The median age of patients included in the Aristocaths study was 8.4 years (range, 1.0–17.6 years) at randomization.

## Discussion

Although trial participation in pediatric oncology treatment protocols is excellent, with accrual rates up to 94 %, accrual in supportive care protocols remains a challenge with accrual rates of less than 50 % [18, 19]. This poor accrual can predominantly be attributed to trial factors: some trial factors cannot be overcome since they are part of the study hypothesis. Study participation by multiple centers can be assisted by the allocation of an initiating center and principal investigator. They can facilitate study implementation through study coordination and provide monetary reimbursements for research personnel. The principal investigator can increase awareness of the trial and elucidate the relevance of the trial’s hypothesis. Physicians’ and parents’ perspectives are based upon perceived benefits and burdens of study participation. Considering the Aristocaths study, parents and physicians will balance the benefit of infection prevention against the burden of 2-h waiting for study locks. Therefore, adequate and tailor-made information is needed to facilitate a well-informed decision by family and child. Also, parents should be offered additional time to make a decision regarding trial participation of their child, especially given the current rapid increase in clinical trials [13].

The necessity and relevance of treatment protocols often need less explanation compared to supportive care protocols since they are considered standard of care by most pediatric oncologists. Nevertheless, morbidity during and after treatment still approaches 40 %, and 1 % of pediatric oncology patients die of infections or other severe complications [20]. Well-designed randomized controlled trials in supportive care are of great importance to improve outcome in pediatric oncology. Unfortunately, many questions concerning supportive care remain unanswered because trials were too small or prematurely stopped because of poor accrual [21–23]. Therefore, explanation by principal investigators alone will not be enough to preserve studies in supportive care. National and international supportive care working groups should increase awareness regarding the need for well-designed randomized controlled trials in supportive care and stimulate accrual when such trials are open.
